# Associations of Ambient Environmental Conditions with Growth and Dissemination of *Staphylococcus epidermidis* on the Surface of Teatcups from Sheep Milking Parlours

**DOI:** 10.3390/bioengineering10010081

**Published:** 2023-01-07

**Authors:** Eleni I. Katsarou, Efthymia Petinaki, George C. Fthenakis

**Affiliations:** 1 Veterinary Faculty, University of Thessaly, 43100 Karditsa, Greece; 2 University Hospital, University of Thessaly, 41110 Larisa, Greece

**Keywords:** humidity, mastitis, sheep, silicone teatcup, *Staphylococcus*, temperature

## Abstract

The growth of two isolates of *Staphylococcus epidermidis* (one that was forming biofilm and one that was not) on new or used teatcups made of silicone for use in milking parlours for sheep, was assessed for 24 h after the application by smearing on the surface of the teatcup. Staphylococci were applied by smearing on an area of 0.0003142 (3.142 × 10^−4^) m^2^ on material obtained from the teatcups and their growth and expansion further on were monitored for 24 h at varying ambient conditions: temperature 21 °C or 31 °C and humidity 60% or 80%. No differences were evident between the two isolates in the frequency of recoveries in any of the conditions tested (*p* > 0.75 for all comparisons). Recovery rates were higher in humidity 80% compared to humidity 60%: 1678/2016 (83.2%) versus 1282/2016 (63.6%) (*p* < 0.0001), and in temperature 31 °C compared to temperature 21 °C: 1525/2016 (75.6%) versus 1435/2016 (71.2%) (*p* = 0.001). Recovery rates were also higher from new teatcups compared to used ones only in humidity 60%: 744/1008 (73.8%) versus 538/1008 (53.4%) (*p* < 0.0001). Humidity 80% was associated with higher speed of linear dissemination of the isolates on teatcup surface compared to humidity 60%: 0.000000640 (6.40 × 10^−7^) m s^−1^ versus 0.000000322 (3.22 × 10^−7^) m s^−1^ (+98.8%) (*p* < 0.0001); no such association was seen with higher temperature: 0.000000509 (5.09 × 10^−7^) m s^−1^ versus 0.000000453 (4.53 × 10^−7^) m s^−1^ for temperature 31 °C and 21 °C (+12.4%) (*p* = 0.29). As part of precision livestock farming, differing approaches can be instituted in accord with varying climatic conditions in different farms, as well as within the same farm with the change of seasons.

## 1. Introduction

Additionally to *Staphylococcus aureus*, which is a frequent and important causal agent of clinical mastitis in sheep, various coagulase-negative species (e.g., *S. epidermidis*, *S. simulans*, *S. chromogenes*) cause subclinical mammary infection. In dairy sheep flocks, the infections of the mammary gland have a significant financial importance [1], because they lead to reduction in milk production, to downgrading of the quality of milk and potentially to rejection of milk in case of administration of antibiotics as part of appropriate treatment. Additional costs incurred as the result of the infection are expenses for purchase of replacement animals and expenses for veterinary care. Mastitis is also an important factor reducing the welfare status of affected sheep [2].

Pathogen transmission can occur during machine-milking of animals [3]. Staphylococci present on the teatcups can enter into the teat duct, when animals are milked; if local defences are compromised, the bacteria may invade the mammary gland and cause an infection. In a previous paper, we described a model developed for the in vitro study of the growth of bacteria on the surface of teatcups [4].

A hypothesis was developed that varying conditions in the environment of a milking parlour (temperature, humidity) may affect the growth of bacteria and their spread on the teatcups. However, there is clear lack of relevant studies and information in the international literature; in a search in the Web of Science platform no relevant publications were found. In general, previous relevant research has focused on the potential to reduce staphylococcal populations on teatcups through correct post-milking cleaning [5] and the potential role of milk residues on teatcups (consequently to incorrect cleaning procedures) [6], as well as on the importance of using disinfectants [7] or other antibacterial products [8]. Hence, the aim of the present work was to assess, in an in vitro study, the potential effects of environmental conditions on the presence and the spread of staphylococci on the surface of teatcups used in milking parlours.

## 2. Materials and Methods

### 2.1. Staphylococcal Isolates

Two isolates of *Staphylococcus epidermidis* were tested in the study. Details on the source of the organisms, multi-locus sequence typing and biofilm production have been presented by Katsarou et al. [9]. In brief, it is noted that these two isolates had been previously recovered from samples of milk collected in Greece, from ewes with subclinical mammary infection [10]. The two organisms were isolated from animals in flocks in which machine milking was carried out. Moreover, both isolates were typed as sequence type (ST) 152 of *S. epidermidis*, as found by carrying out multi-locus sequence typing (MLST). One isolate was forming biofilm (isolate A), whilst the other was not (isolate B) [9].

### 2.2. Test Material

New and used teatcups manufactured for use in milking parlours for sheep (silicone) were used as test material. Brand-new teatcups (Flaco; J Delgado, Ciudad Real, Spain) were purchased from an agricultural merchant. Used teatcups, of the same brand as the new ones, were obtained, through the same agricultural merchant, from a local sheep farm, after these had been replaced in the milking parlour at the end of a milking season, subsequently to a use of approximately 4500 milkings.

Out of the tubular shaft part of each teatcup (i.e., the main part of the teatcup, that extends downwardly from the upper tubular shaft portion and leads to the more narrow milk conduit), a piece of material was cut. The piece was square, with dimensions 8 cm × 8 cm.

**Figure 1:**
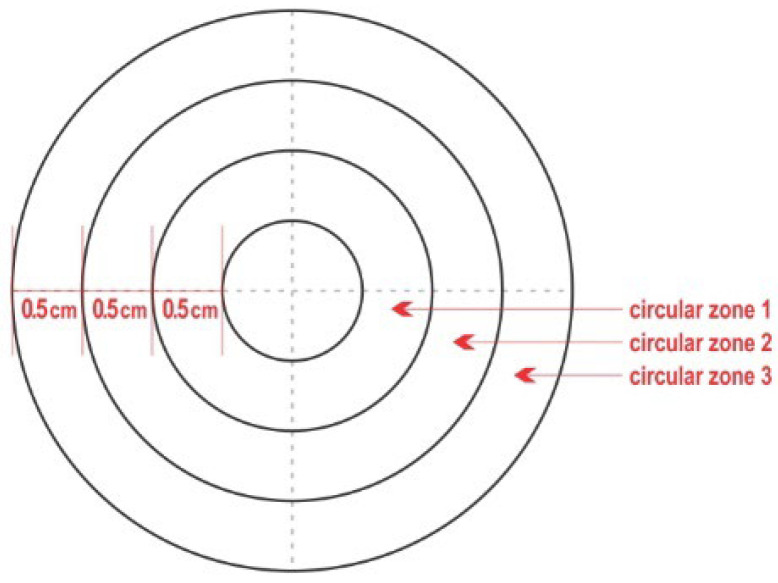
Schematic presentation of the polar chart-type complex that was created on the material obtained from a teatcup [4].

The pieces of teatcup material were pinned on a wooden board, thus remaining stretched and facilitating all technical work. On the middle of each piece of teatcup material, a complex like a polar chart-type (‘complex’) was designed. The complex included four concentric circles, with diametres 1 cm, 2 cm, 3 cm, 4 cm, and thus referred to an inner circle and three outer zones (Figure 1), as described in detail by Katsarou et al. [4]. These zones, from the inner to the middle one to the outermost, were termed ‘circular zone 1′, ‘circular zone 2′ and ‘circular zone 3′, respectively. Then, the inner circle and each of the three circular zones (i.e., the entire complex) were divided into four equal quadrants by drawing two diametres at an angle of 90° between them. That way, in total, 16 quadrants were created on each complex. This in vitro model has been described in detail and validated by Katsarou et al. [4].

### 2.3. Experimental Work

The two bacterial isolates (*S. epidermidis*) were cultured on Columbia blood agar and then seeded into Tryptic Soy broth (BioMerieux, Marcy-l’-Étoile, France) for aerobic incubation at 37 °C for 6 h. After mixing the bacterial suspension in a Vortex equipment (Velp Scientifica, Usmate, Italy), a swab (single cotton tip on a 10 cm wooden handle, sterile, individually packed) (Shanghai International Corporation, Hamburg, Germany) was dipped into the broth for 3 s; excess fluid was drained and the swab was withdrawn. The swab was smeared on the piece of teatcup material as described in minute detail by Katsarou et al. [4], specifically on the inner circle of the complex to cover this circle completely. The concentration of colony-forming-units (c.f.u.) in the broth varied from 4.41 × 10^9^ to 8.93 × 10^10^ c.f.u. mL^−1^; the established technique of Miles and Misra [11] was employed for this calculation.

Samplings (which referred to touching the surface of the test material with the side of the cotton tip of a sterile swab) were performed every 3 h up to 18 h and then at 24 h [4]. The procedure was performed on each of the 12 quadrants in the three outer circular zones (these were described above, Section 2.2.). On each procedure, two samples were obtained from each quadrant, by using on each occasion a new sterile swab [4].

The swab samples obtained during the above procedure were cultured on 5% sheep blood agar and *Staphylococcus* selective medium (mannitol agar) in duplicate. All the media were incubated at 37 °C in aerobic environment for a period of 48 h; if no growth was seen at that time, they were reincubated for a further 24 h. Bacterial isolation and initial identification were carried out using established techniques [12,13]. Detection of at least one staphylococcal colony with a similar morphology to that of the colonies of the challenge isolates (*S. epidermidis*) from at least one of the two swab samples obtained from a quadrant on each sampling occasion (i.e., if bacteria were isolated from one swab of the four used for sampling and cultured) was deemed to confirm presence of the bacteria on the test material, specifically on the quadrant of the complex from where the swab sample was collected.

### 2.4. Ambient Conditions

After bacterial application by smearing, the board with the pieces of test material was maintained at temperature 21.0 °C or 31.0 °C and humidity 60% or 80%. Ambient temperature and humidity were maintained by means of an air-conditioning unit and a humidifier, functioning throughout each experiment.

For each isolate, triplicates were performed on each environmental condition and for each type of material (new or old teatcup) assessed. Uninoculated complexes, with no bacteria smeared thereon, were also included in each assessment as controls.

### 2.5. Data Management and Analysis

As the model employed had been validated by Katsarou et al. [4], similar data management was applied, in order to maintain compliance with that model. The area οf each of zone 1, zone 2 and zone 3 was 0.0009424 (9.424 × 10^−4^) m^2^, 0.0015708 (15.708 × 10^−4^) m^2^ and 0.0021990 (21.990 × 10^−4^) m^2^, respectively. Thus, the area of each of the quadrants in the respective zones were 0.0002356 (2.356 × 10^−4^) m^2^, 0.0003927 (3.927 × 10^−4^) m^2^ and 0.0005498 (5.498 × 10^−4^) m^2^. Thereafter, the length of time required for the full coverage of each quadrant in circular zone 1 and circular zone 2 was calculated as detailed previously [4]; the average speed of the linear dissemination of an isolate through a complex was also calculated as before [4], as the average speed in the four quadrants (i.e., in the four directions). Finally, the total surface of the complex that had been covered 12 h after application by smearing was considered.

Data were entered into Microsoft Excel and analyzed using SPSS v. 21 (IBM Analytics, Armonk, NY, USA). Basic descriptive analysis was performed. Frequencies were evaluated by use of Pearson chi-square test or Fisher exact test, as appropriate. Results of the speed of linear dissemination and surface covered were compared between material (new or old teatcups) and between conditions (high or low temperature, high or low humidity) by using the Kruskal–Wallis test. Statistical significance was defined at *p* < 0.05.

## 3. Results

### 3.1. Frequency of Staphylococcal Recoveries

No bacteria were recovered from any of the swab samples obtained at the start of each trial, before applying (smearing) the staphylococcal isolates on the teat material.

**Figure 2:**
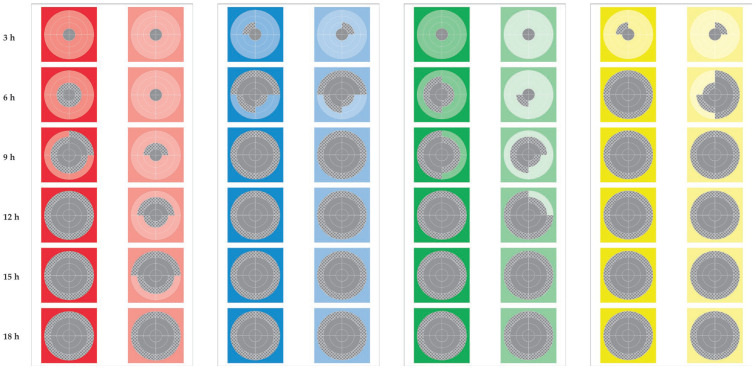
Schematic presentation of the gradual growth of *S. epidermidis* on eight polar charts on teatcup material. (Legend; hours shown in the left refer to times after application of the *Staphylococcus* by smearing; red-coloured stripes: temperature 21 °C, humidity 60%; blue-coloured stripes: temperature 21 °C, humidity 80%; green-coloured stripes: temperature 31 °C, humidity 60%; yellow-coloured stripes: temperature 31 °C, humidity 80%; dark-coloured stripes: new teatcups, lightly-coloured stripes: used teatcups; grey motif pattern denotes the presence of *S. epidermidis* on a quadrant, grey full pattern denotes the full coverage of a quadrant with *S. epidermidis*).

In all trials, after application by smearing, we recovered staphylococci consistently from the swab samples collected from the inner circle of the complex on all sampling occasions (3 h to 24 h post-application) (Figure 2). This was consistently found for all triplicate samples examined, for both isolates, for the two types of test material (new or used teatcups) and for both sets of environmental conditions (high or low temperature, high or low humidity) (*p* > 0.95 for all comparisons between triplicate measurements in the various assessments). In contrast, we did not recover staphylococci at any sampling point from any complex, on which no staphylococci had been applied (*p* < 0.0001 for all comparisons versus complexes on which staphylococci were smeared). Differences in overall recovery rates between successive sampling points were significant for up to 18 h after application by smearing (*p* < 0.0005 for all comparisons).

We did not find differences in the frequencies of bacterial recovery between the two isolates: cumulative recoveries were 1483/2016 (73.6%) for isolate A and 1477/2016 (73.3%) for isolate B (*p* = 0.83). Also, we did not find significant differences in recovery rates between the two isolates, when assessing separately the three circular zones for recoveries between the two isolates (*p* > 0.75 for all comparisons) (Tables S1–S4).

We found clear evidence that recoveries were more frequent from new teatcups compared to used ones: 1593/2016 (79.0%) versus 1367/2016 (67.8%) (*p* < 0.0001). We found this significant difference also in the recovery rates from the two types of teatcups, when these were considered separately for recoveries in each of the three circular zones (*p* < 0.0001). However, when we assessed recoveries separately in the environmental conditions created, this difference was evident in humidity 60% (*p* < 0.0001 for all comparisons), but not in humidity 80% (*p* > 0.33 for all comparisons) (Table 1). When we assessed separately the three circular zones for recoveries, significant differences in recoveries from new and used teatcups were seen in humidity 60% (*p* < 0.0001 for all comparisons), but not in humidity 80% (*p* > 0.18 for all comparisons).

In general, we found that recovery rates were higher in humidity 80% compared to humidity 60%: 1678/2016 (83.2%) versus 1282/2016 (63.6%) (*p* < 0.0001) (Table 1). The same differences were evident when the three circular zones were assessed separately for recoveries (*p* < 0.0001).

Also, we found that recovery rates were higher in temperature 31 °C compared to temperature 21 °C: 1525/2016 (75.6%) versus 1435/2016 (71.2%) (*p* = 0.001) (Table 2). However, when the three circular zones were assessed separately for recoveries, only tendencies for significance were noted: *p* = 0.052, *p* = 0.058 and *p* = 0.074 for recoveries from circular zones 1, 2 and 3, respectively.

Finally, we did not find any differences in the frequencies of recovery of staphylococci among the triplicate assays during the evaluation of the same isolate tested on the same material (*p* > 0.95).

### 3.2. Speed of Staphylococcal Dissemination

There was no difference in the speed of dissemination between the two isolates overall: 0.000000491 (4.91 × 10^−7^) m s^−1^ and 0.000000471 (4.71 × 10^−7^) m s^−1^, respectively (*p* = 0.71). The speed of dissemination was clearly higher in new teatcups: 0.000000373 (3.73 × 10^−7^) m s^−1^ versus 0.000000271 (2.71 × 10^−7^) m s^−1^ in used ones (*p* < 0.0001) in 60% humidity, whilst no such difference was seen in 80% humidity: 0.000000664 (6.64 × 10^−7^) m s^−1^ versus 0.000000616 m (6.16 × 10^−7^) s^−1^, respectively (*p* = 0.23).

Higher humidity (80%) was associated with higher speed compared to 60% humidity: 0.000000640 (6.40 × 10^−7^) m s^−1^ versus 0.000000322 (3.22 × 10^−7^) m s^−1^ (+98.8%), respectively (*p* < 0.0001). In contrast, no association of temperature was seen with significantly higher speed of dissemination: 0.000000509 (5.09 × 10^−7^) m s^−1^ versus 0.000000453 (4.53 × 10^−7^) m s^−1^ (+12.4%), for temperature 31 °C and 21 °C respectively (*p* = 0.29).

### 3.3. Teatcup Surface Covered by Staphylococci

**Figure 3:**
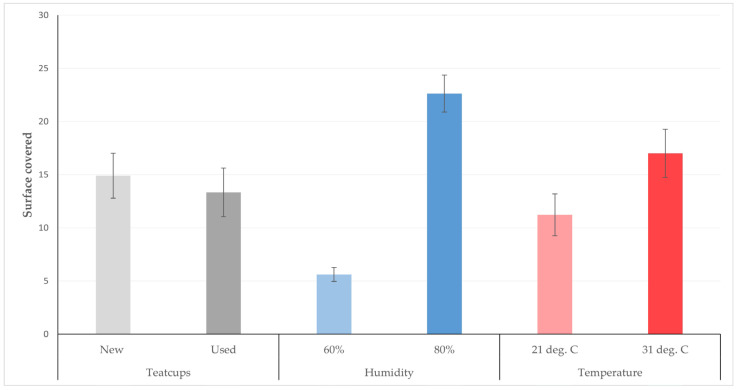
Average surface (cm^2^) of teatcups covered by *S. epidermidis* in 12 h after application by smearing on teatcups (Legend; deg. C: °C).

**Figure 4:**
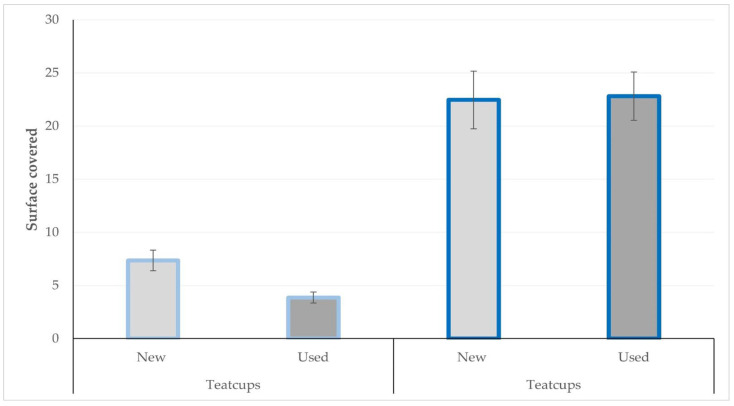
Average surface (cm^2^) of teatcups covered by *S. epidermidis* in 12 h after application by smearing on teatcups (Legend; bars with light blue outline: humidity = 60%; bars with dark blue outline: humidity = 80%).

The surface of the teatcup material considered to have been covered within 12 h after application by smearing in the various ambient conditions for new and used teatcups, are presented in Table 3 and in Figure 3 and Figure 4.

## 4. Discussion

*S. epidermidis* can cause clinical and subclinical mammary infection in sheep [14,15], thus it is interesting to study potential for dissemination at the milking equipment level, given that a significant proportion of mammary infections takes place during milking. The present results provide evidence that dissemination of the organism on milking teatcups can be dependent on ambient humidity: higher humidity (80% versus 60%) promoted rapid dissemination of the pathogen, which quickly covered an increased surface of the teatcup. To note that in previous studies, high humidity within hospitals buildings was found to result in longer survival of the organism [16]; moreover, high humidity also afforded an alleviating effect to damage to the bacteria by ultraviolet light [17].

Staphylococci adhere to surfaces, grow and spread by means of various factors. In increased humidity, they may float on the moisture available on the surface (i.e., the teatcup) and disseminate quickly [18,19]. Increased ambient humidity may minimize the adhesive forces holding staphylococcal microcolonies together, that way ejecting cells more frequently [19], which contributes to the higher speed of bacterial dissemination.

The reduced growth of the bacteria on the used teatcups was not expected. A possible explanation for this might be that the fissures and cracks present on used teatcups (which have occurred as result of material fatigue due to cyclic loading [20]), increase the total surface of used teatcups (compared to new teatcups, in which the surface is intact), hence bacteria needed to cover a larger surface. Nevertheless, the bacterial loads in used teatcups could likely be higher ultimately, because these cracks and fissures on the teatcup surface would also contain bacteria, additionally to those on the intact surface of those teatcups. Indeed, the amount of staphylococci adhered onto coarse material can be higher than that on fine surfaces [21].

Formation of biofilm by one of the isolates evaluated did not seem to provide an advantage in dissemination of the staphylococci on the surface of the teatcup. Through formation of biofilm, bacteria attach on surfaces and produce extracellular polymers, which enhance their attachment thereon and the formation of matrices [22]. In the present study, two different staphylococcal isolates were included: one was an isolate not forming biofilm, whilst the other was a strong biofilm-former; there were no differences in the other properties of the two isolates: they belonged to the same ST (ST152), they were fully susceptible to antibiotics, they had been recovered from farms using machine-milking. This lack of effect is compatible with ‘darting’, which is a phenomenon by which the dissemination of *S. epidermidis* occurs [19,23]; during darting, dissemination of bacterial colonies takes place by ejection, not by gradual attachment onto the surface. Biofilm-formation can be important for the survival of these bacteria during cleaning, as found in a previous study, where the majority of staphylococci (87%) isolated from teatcups from milking parlours were biofilm-formers [24], but evidently not for their gradual dissemination on the teatcups. These results are fully aligned with those of our initial study, performed on material from brand-new teatcups, in which also no differences were found in the dissemination of these two isolates [4].

The findings suggest that, in field conditions, differing climatic conditions can pose varying challenges in the management of milking parlours in farms. As part of precision livestock farming, differing approaches can be instituted in accord with varying climatic conditions in different farms, as well as within the same farm with change of seasons. Within a wider viewpoint, it is noted that climatic changes may lead to effects in micro-environments, e.g., within a milking parlour, and have relevant effects in pathogen dissemination. In a similar context, it is noted that humidity has been recognized as a risk factor for staphylococcal infections in orthopaedic prosthetic devices in the tropical part of Australia [25], which further indicates the importance of climatic conditions in bacterial infections.

The study presents for the first time internationally that climatic factors may affect the bacterial numbers and their speed of dissemination on teatcups from milking parlours. Increased number of bacterial on teatcups increase the risk for mammary infections and consequently mastitis to animals milked in those parlours [3]. Previous studies have indicated that increased precipitation (which leads to increased humidity, a factor found to be important for bacterial growth on teatcups in the present study) can be used to predict increased incidence of mastitis [26]. Thusfar, we have hypothesized that climatic factors could affect the animal response to invading bacteria; for example, Vasileiou et al. [27] have suggested that increased environmental temperatures (another factor identified in the present study to be important for bacterial growth on teatcups) could lead in impaired leucocyte function of animals, which would facilitate development of mastitis. The present results indicate that also environmental factors clearly affect the bacterial populations on the teatcups in milking parlours, i.e., at very close proximity to mammary glands, hence increasing the risk for infections.

The results the study can be used to scientifically update the relevant knowledge about conditions in milking parlours, because indeed conditions change as the results of different dynamics or interactions prevailing in there change, according to time of year, technological changes and bacterial evolution.

## Figures and Tables

**Table 1:** Recovery of two bacterial isolates (*Staphylococcus epidermidis*) after application on teatcups of milking parlour for sheep, in accord with type of material and ambient humidity.

Time after Application by Smearing	Total Recoveries		
	New Teatcups	Used Teatcups	*p*
Humidity 60%			
3 h	1/144	0/144	0.32
6 h	58/144	2/144	<0.0001
9 h	109/144	30/144	<0.0001
12 h	144/144	86/144	<0.0001
15 h	144/144	132/144	0.0004
18 h	144/144	144/144	n/a
24 h	144/144	144/144	n/a
Total	744/1008	538/1008	<0.0001
Humidity 80%			
3 h	5/144	10/144	0.18
6 h	124/144	100/144	0.0007
9 h	144/144	143/144	0.32
12 h	144/144	144/144	n/a
15 h	144/144	144/144	n/a
18 h	144/144	144/144	n/a
24 h	144/144	144/144	n/a
Total	849/1008	829/1008	0.23
Grand total	1593/2016	1367/2016	<0.0001

**Table 2:** Recovery of two bacterial isolates (*S. epidermidis*) after application on teatcups of milking parlour for sheep, in accord with type of material and ambient temperature.

Time after Application by Smearing	Total Recoveries		
	New Teatcups	Used Teatcups	*p*
Temperature 21 °C			
3 h	1/144	3/144	0.56
6 h	82/144	46/144	<0.0001
9 h	122/144	81/144	<0.0001
12 h	144/144	104/144	<0.0001
15 h	144/144	132/144	0.0004
18 h	144/144	144/144	n/a
24 h	144/144	144/144	n/a
Total	781/1008	654/1008	<0.0001
Temperature 31 °C			
3 h	5/144	7/144	0.56
6 h	100/144	56/144	<0.0001
9 h	131/144	92/144	<0.0001
12 h	144/144	126/144	<0.0001
15 h	144/144	144/144	n/a
18 h	144/144	144/144	n/a
24 h	144/144	144/144	n/a
Total	812/1008	713/1008	<0.0001
Grand total	1593/2016	1367/2016	<0.0001

**Table 3:** Surface (cm^2^) considered to have been covered in 12 h after application by smearing on new or used teatcups by *S. epidermidis*, in accord with ambient temperature and humidity.

Ambient Conditions ^1^	New Teatcups	Used Teatcups	*p*
21 °C, 60%	5.048 ± 0.930 ^a,b^	2.588 ± 0.459 ^a,b^	0.039
21 °C, 80%	17.170 ± 3.953 ^a,c^	20.119 ± 3.070 ^a,c^	0.57
31 °C, 60%	9.682 ± 1.061 ^d^	5.145 ± 0.566 ^c,d^	0.004
31 °C, 80%	27.730 ± 2.460 ^b,c,d^	25.491 ± 3.271 ^b,d^	0.60
*p*	<0.0001	<0.0001	

^1^ Temperature, humidity, respectively. ^a,b,c,d^ Within the same column, differences are significant between figures marked with the same superscript (*p* < 0.03 for left column, *p* < 0.01 for right column).

## Data Availability

The data associated with this manuscript are provided within the manuscript or at the Supplementary Materials.

## Supplementary Materials

The following supporting information can be downloaded at: https://www.mdpi.com/article/10.3390/bioengineering10010081/s1, Table S1: Detailed findings of the recovery of two bacterial isolates (*S. epidermidis*) from the surface of teatcups made of silicone (for use in sheep), in temperature 21 °C and humidity 60%; Table S2: Detailed findings of the recovery of two bacterial isolates (*S. epidermidis*) from the surface of teatcups made of silicone (for use in sheep), in temperature 21 °C and humidity 80%; Table S3: Detailed findings of the recovery of two bacterial isolates (*S. epidermidis*) from the surface of teatcups made of silicone (for use in sheep), in temperature 31 °C and humidity 60%; Table S4: Detailed findings of the recovery of two bacterial isolates (*S. epidermidis*) from the surface of teatcups made of silicone (for use in sheep), in temperature 31 °C and humidity 80%.
