# Supplementary material for: Associations of Ambient Environmental Conditions with Growth and Dissemination of Staphylococcus epidermidis on the Surface of Teatcups from Sheep Milking Parlours
Source: Bioengineering (Basel). 2023 Jan 7;10(1):81. doi: 10.3390/bioengineering10010081 (PMC9855130; doi:10.3390/bioengineering10010081)
Supplement: Supplementary file 1 [file bioengineering-10-00081-s001.zip › bioengineering-2092692-supplementary.pdf]

Table S1. Detailed findings of the recovery of two bacterial isolates (*S. epidermidis*) from the surface of teatcups made of silicone (for use in sheep), in temperature 21 °C and humidity 60%.

Time after application by smearing	Recoveries from circular zone 1	Recoveries from circular zone 2	Recoveries from circular zone 3	Total recoveries	Recoveries from circular zone 1	Recoveries from circular zone 2	Recoveries from circular zone 3	Total recoveries
	**New teatcups**				**Used teatcups**			
Isolate A (biofilm-forming)								
3 h	0/12	0/12	0/12	0/36	0/12	0/12	0/12	0/36
6 h	11/12	1/12	0/12	12/36	0/12	0/12	0/12	0/36
9 h	12/12	12/12	2/12	26/36	6/12	0/12	0/12	6/36
12 h	12/12	12/12	12/12	36/36	11/12	5/12	2/12	18/36
15 h	12/12	12/12	12/12	36/36	12/12	12/12	7/12	31/36
18 h	12/12	12/12	12/12	36/36	12/12	12/12	12/12	36/36
24 h	12/12	12/12	12/12	36/36	12/12	12/12	12/12	36/36
Totals	71/84	61/84	50/84	182/252	53/84	41/84	33/84	127/252
Isolate B (non biofilm-forming)								
3 h	0/12	0/12	0/12	0/36	0/12	0/12	0/12	0/36
6 h	11/12	1/12	0/12	12/36	0/12	0/12	0/12	0/36
9 h	12/12	11/12	1/12	24/36	4/12	0/12	0/12	4/36
12 h	12/12	12/12	12/12	36/36	9/12	4/12	1/12	14/36
15 h	12/12	12/12	12/12	36/36	11/12	9/12	9/12	29/36
18 h	12/12	12/12	12/12	36/36	12/12	12/12	12/12	36/36
24 h	12/12	12/12	12/12	36/36	12/12	12/12	12/12	36/36
Totals	71/84	60/84	49/84	180/252	48/84	37/84	34/84	119/252

Table S2. Detailed findings of the recovery of two bacterial isolates (*S. epidermidis*) from the surface of teatcups made of silicone (for use in sheep), in temperature 21 °C and humidity 80%.

Time after application by smearing	Recoveries from circular zone 1	Recoveries from circular zone 2	Recoveries from circular zone 3	Total recoveries	Recoveries from circular zone 1	Recoveries from circular zone 2	Recoveries from circular zone 3	Total recoveries
	**New teatcups**				**Used teatcups**			
Isolate A (biofilm-forming)								
3 h	1/12	0/12	0/12	1/36	1/12	0/12	0/12	1/36
6 h	12/12	10/12	7/12	29/36	11/12	10/12	6/12	27/36
9 h	12/12	12/12	12/12	36/36	12/12	12/12	12/12	36/36
12 h	12/12	12/12	12/12	36/36	12/12	12/12	12/12	36/36
15 h	12/12	12/12	12/12	36/36	12/12	12/12	12/12	36/36
18 h	12/12	12/12	12/12	36/36	12/12	12/12	12/12	36/36
24 h	12/12	12/12	12/12	36/36	12/12	12/12	12/12	36/36
Totals	73/84	70/84	67/84	210/252	72/84	70/84	66/84	208/252
Isolate B (non biofilm-forming)								
3 h	0/12	0/12	0/12	0/36	2/12	0/12	0/12	2/36
6 h	12/12	9/12	8/12	29/36	10/12	5/12	4/12	19/36
9 h	12/12	12/12	12/12	36/36	12/12	12/12	11/12	35/36
12 h	12/12	12/12	12/12	36/36	12/12	12/12	12/12	36/36
15 h	12/12	12/12	12/12	36/36	12/12	12/12	12/12	36/36
18 h	12/12	12/12	12/12	36/36	12/12	12/12	12/12	36/36
24 h	12/12	12/12	12/12	36/36	12/12	12/12	12/12	36/36
Totals	72/84	69/84	68/84	209/252	72/84	65/84	63/84	200/252

Table S3. Detailed findings of the recovery of two bacterial isolates (*S. epidermidis*) from the surface of teatcups made of silicone (for use in sheep), in temperature 31 ^{o}C and humidity 60%.

Time after application by smearing	Recoveries from circular zone 1	Recoveries from circular zone 2	Recoveries from circular zone 3	Total recoveries	Recoveries from circular zone 1	Recoveries from circular zone 2	Recoveries from circular zone 3	Total recoveries
	New teatcups				Used teatcups			
Isolate A (biofilm-forming)								
3 h	0/12	0/12	0/12	0/36	0/12	0/12	0/12	0/36
6 h	12/12	3/12	2/12	17/36	0/12	0/12	0/12	0/36
9 h	12/12	12/12	4/12	28/36	10/12	0/12	0/12	10/36
12 h	12/12	12/12	12/12	36/36	12/12	10/12	4/12	26/36
15 h	12/12	12/12	12/12	36/36	12/12	12/12	12/12	36/36
18 h	12/12	12/12	12/12	36/36	12/12	12/12	12/12	36/36
24 h	12/12	12/12	12/12	36/36	12/12	12/12	12/12	36/36
Totals	72/84	63/84	54/84	189/252	58/84	46/84	40/84	144/252
Isolate B (non biofilm-forming)								
3 h	1/12	0/12	0/12	1/36	0/12	0/12	0/12	0/36
6 h	12/12	4/12	2/12	18/36	2/12	0/12	0/12	2/36
9 h	12/12	12/12	6/12	30/36	8/12	2/12	0/12	10/36
12 h	12/12	12/12	12/12	36/36	12/12	11/12	5/12	28/36
15 h	12/12	12/12	12/12	36/36	12/12	12/12	12/12	36/36
18 h	12/12	12/12	12/12	36/36	12/12	12/12	12/12	36/36
24 h	12/12	12/12	12/12	36/36	12/12	12/12	12/12	36/36
Totals	73/84	64/84	56/84	193/252	58/84	49/84	41/84	148/252

Table S4. Detailed findings of the recovery of two bacterial isolates (*S. epidermidis*) from the surface of teatcups made of silicone (for use in sheep), in temperature 31 ^{o}C and humidity 80%.

Time after application by smearing	Recoveries from circular zone 1	Recoveries from circular zone 2	Recoveries from circular zone 3	Total recoveries	Recoveries from circular zone 1	Recoveries from circular zone 2	Recoveries from circular zone 3	Total recoveries
	New teatcups				Used teatcups			
Isolate A (biofilm-forming)								
3 h	2/12	0/12	0/12	2/36	3/12	0/12	0/12	3/36
6 h	12/12	12/12	10/12	34/36	12/12	8/12	4/12	24/36
9 h	12/12	12/12	12/12	36/36	12/12	12/12	12/12	36/36
12 h	12/12	12/12	12/12	36/36	12/12	12/12	12/12	36/36
15 h	12/12	12/12	12/12	36/36	12/12	12/12	12/12	36/36
18 h	12/12	12/12	12/12	36/36	12/12	12/12	12/12	36/36
24 h	12/12	12/12	12/12	36/36	12/12	12/12	12/12	36/36
Totals	74/84	72/84	70/84	216/252	75/84	68/84	64/84	207/252
Isolate B (non biofilm-forming)								
3 h	2/12	0/12	0/12	2/36	4/12	0/12	0/12	4/36
6 h	12/12	11/12	9/12	32/36	12/12	11/12	7/12	30/36
9 h	12/12	12/12	12/12	36/36	12/12	12/12	12/12	36/36
12 h	12/12	12/12	12/12	36/36	12/12	12/12	12/12	36/36
15 h	12/12	12/12	12/12	36/36	12/12	12/12	12/12	36/36
18 h	12/12	12/12	12/12	36/36	12/12	12/12	12/12	36/36
24 h	12/12	12/12	12/12	36/36	12/12	12/12	12/12	36/36
Totals	74/84	71/84	69/84	214/252	76/84	71/84	67/84	214/252
